# Independence of Lipoprotein(a) and Low-Density Lipoprotein Cholesterol–Mediated Cardiovascular Risk: A Participant-Level Meta-Analysis

**DOI:** 10.1161/CIRCULATIONAHA.124.069556

**Published:** 2024-11-04

**Authors:** Harpreet S. Bhatia, Simon Wandel, Peter Willeit, Anastasia Lesogor, Keith Bailey, Paul M. Ridker, Paul Nestel, John Simes, Andrew Tonkin, Gregory G. Schwartz, Helen Colhoun, Christoph Wanner, Sotirios Tsimikas

**Affiliations:** 1 Division of Cardiology, Department of Medicine, University of California, San Diego, La Jolla (H.S.B., S.T.).; 2 Novartis Pharma AG, Basel, Switzerland (S.W., A.L., K.B.).; 3 Department of Medical Statistics, Informatics and Health Economics at the Medical University of Innsbruck, Austria (P.W.).; 4 Brigham and Women’s Hospital, Harvard Medical School, Boston, MA (P.M.R.).; 5 Baker Heart and Diabetes Institute, Melbourne, Victoria, Australia (P.N.).; 6 NHMRC Clinical Trials Centre, University of Sydney, NSW, Australia (J.S.).; 7 School of Public Health and Preventive Medicine, Monash University, Melbourne, Victoria, Australia (A.T.).; 8 Division of Cardiology, Rocky Mountain Regional VA Medical Center and University of Colorado School of Medicine, Aurora (G.G.S.).; 9 MRC Human Genetics Unit, Centre for Genomic and Experimental Medicine, MRC Institute of Genetics & Molecular Medicine, Edinburgh, UK (H.C.).; 10 Division of Nephrology, Department of Internal Medicine and Comprehensive Heart Failure Centre, University Hospital of Würzburg, Germany (C.W.).

**Keywords:** cholesterol, heart disease risk factors, lipoprotein(a), lipoproteins, LDL

## Abstract

**BACKGROUND:**

Low-density lipoprotein cholesterol (LDL-C) and lipoprotein(a) (Lp[a]) levels are independently associated with atherosclerotic cardiovascular disease (ASCVD). However, the relationship between Lp(a) level, LDL-C level, and ASCVD risk at different thresholds is not well defined.

**METHODS:**

A participant-level meta-analysis of 27 658 participants enrolled in 6 placebo-controlled statin trials was performed to assess the association of LDL-C and Lp(a) levels with risk of fatal or nonfatal coronary heart disease events, stroke, or any coronary or carotid revascularization (ASCVD). The multivariable-adjusted association between baseline Lp(a) level and ASCVD risk was modeled continuously using generalized additive models, and the association between baseline LDL-C level and ASCVD risk by baseline Lp(a) level by Cox proportional hazards models with random effects. The joint association between Lp(a) level and statin-achieved LDL-C level with ASCVD risk was evaluated using Cox proportional hazards models.

**RESULTS:**

Compared with an Lp(a) level of 5 mg/dL, increasing levels of Lp(a) were log-linearly associated with ASCVD risk in statin- and placebo-treated patients. Among statin-treated individuals, those with Lp(a) level >50 mg/dL (≈125 nmol/L) had increased risk across all quartiles of achieved LDL-C level and absolute change in LDL-C level. Even among those with the lowest quartile of achieved LDL-C level (3.1–77.0 mg/dL), those with Lp(a) level >50 mg/dL had greater ASCVD risk (hazard ratio, 1.38 [95% CI, 1.06–1.79]) than those with Lp(a) level ≤50 mg/dL. The greatest risk was observed with both Lp(a) level >50 mg/dL and LDL-C level in the fourth quartile (hazard ratio, 1.90 [95% CI, 1.46–2.48]).

**CONCLUSIONS:**

These findings demonstrate the independent and additive nature of Lp(a) and LDL-C levels for ASCVD risk, and that LDL-C lowering does not fully offset Lp(a)-mediated risk.

**Clinical Perspective**

**What Is New?**

- Lipoprotein(a) (Lp[a]) level is associated with cardiovascular risk continuously and independently of low-density lipoprotein cholesterol (LDL-C) level.
- Elevated cardiovascular risk persists when Lp(a) level is elevated across strata of achieved LDL-C level and change in LDL-C level, even with the lowest levels of achieved LDL-C.
- The greatest risk was observed with the highest levels of both Lp(a) and LDL-C.

**What Are the Clinical Implications?**

- Lp(a) and LDL-C levels are independent and additive for cardiovascular risk.
- Reduction in LDL-C level cannot fully offset Lp(a)-mediated risk.
- There is a need for targeted therapy for both LDL-C and Lp(a) levels.
- More widespread Lp(a) testing is needed to identify patients at risk.

Low-density lipoprotein cholesterol (LDL-C) and lipoprotein(a) (Lp[a]) are independently associated with greater risk for atherosclerotic cardiovascular disease (ASCVD).^1–3^ Management of elevated LDL-C levels, particularly in the context of elevated ASCVD risk, has been well established in clinical practice guidelines, with statins as first-line therapy, followed by ezetimibe and proprotein convertase subtilisin/kexin type 9 inhibitors (PCSK9i) in those who do not achieve prespecified goals.^1^ However, the understanding of the optimal management of patients with elevated Lp(a) levels, particularly in the context of LDL-C, is uncertain.

American College of Cardiology/American Heart Association guidelines recommend use of elevated Lp(a) level as a risk enhancer for overall risk assessment,^1^ which primarily guides the use of statin and other lipid-lowering therapies. However, the European Society of Cardiology/European Atherosclerosis Society, Canadian Cardiovascular Society, and recent National Lipid Association guidelines recommend measuring Lp(a) level once in all adults, and these guidelines and the European Atherosclerosis Society consensus statement recommend addressing LDL-C levels more aggressively when the Lp(a) level is elevated to attempt to offset Lp(a)-mediated risk.^3–6^ However, previous studies have suggested that ASCVD risk is elevated in association with elevated Lp(a) level, even when the LDL-C level is relatively low^7,8^ or despite a background of moderate to high-intensity statin therapy.^9–11^

Statin use is associated with reduced ASCVD risk, but statins do not lower Lp(a) levels and may increase them, and therefore may not modify an important contributor to risk.^11–15^ Conversely, the benefit of PCSK9i, which lower both LDL-C and Lp(a) levels, appears to be augmented in those with elevated Lp(a) level.^9,10^ The Cholesterol Treatment Trialists empiric formula^16^ relates absolute reduction in LDL-C level to relative reduction in cardiovascular risk under statin treatment, but it was derived without consideration of Lp(a) levels. Based on these considerations, it is possible that the canonical Cholesterol Treatment Trialists relationship actually reflects an aggregate of a family of relationships that differ according to the prevailing level of Lp(a).

With the advent of targeted therapies for Lp(a),^17^ it is important to understand the interaction between LDL-C and Lp(a) for ASCVD risk and the corresponding therapeutic implications. A participant-level meta-analysis of 6 placebo-controlled statin trials was performed to determine the continuous association between baseline Lp(a) level and ASCVD risk in placebo- or statin-treated patients and the association of risk for ASCVD events with elevated Lp(a) level across statin-achieved LDL-C levels.

## METHODS

### Clinical Trials

Six randomized, placebo-controlled trials of statin therapy from the Lipoprotein(a) Studies Collaboration were included in this analysis. Participants from the 4D Study (Die Deutsche Diabetes Dialyse),^18^ 4S (Scandinavian Simvastatin Survival Study),^19^ CARDS (Collaborative Atorvastatin Diabetes Study),^20^ JUPITER (Justification for the Use of Statins in Prevention: An Intervention Trial Evaluating Rosuvastatin),^21^ LIPID (Long-Term Intervention With Pravastatin in Ischaemic Disease),^22^ and MIRACL (Myocardial Ischemia Reduction With Aggressive Cholesterol Lowering)^23^ were included. The designs of these studies and methods of Lp(a) measurement have been summarized previously. Ethical approval and informed consent were obtained as part of each study.^11^ Requests to access the data set from qualified researchers trained in human participant confidentiality protocols may be sent to individual trial investigators. Participants with baseline Lp(a) and LDL-C measurements and follow-up for cardiovascular disease outcomes were included.

For all participants, baseline demographic characteristics, cardiovascular risk factors, vital signs, and lipid measurements were collected. The primary ASCVD end point was defined as the incidence of fatal or nonfatal coronary heart disease, fatal or nonfatal stroke, or any coronary or carotid revascularization. Of note, 5 of these 6 trials (all but MIRACL) had median follow-up exceeding 2.3 years, and 5 of the 6 trials (all but 4D) demonstrated a cardiovascular benefit of statin treatment.

### Statistical Analysis

We performed a participant-level meta-analysis of individuals enrolled in 6 trials. For all analyses, Lp(a) levels were log-transformed. Harmonization of Lp(a) measurements from each study has been described previously.^11^ Baseline characteristics are presented as mean (SD), median (interquartile range), or n (%).

First, we evaluated the independent, continuous associations between baseline Lp(a) and LDL-C levels in multivariable adjusted Cox proportional hazards models with study-specific random effects. A continuous assessment of Lp(a) was used, as previous studies have suggested a continuous association between Lp(a) and risk.^24^ The models included both lipoproteins, were adjusted for the covariates of age, sex, history of vascular disease, diabetes, smoking, systolic blood pressure (SBP), and baseline high-density lipoprotein cholesterol (HDL-C) level, and were stratified by trial and treatment assignment. We also evaluated the multiplicative interaction between baseline Lp(a) and LDL-C in these models. In a sensitivity analysis, we evaluated this interaction by sex and age (less than or equal to and greater than the median age of 63 years) as well. Given the association between lower Lp(a) level and diabetes, we performed a sensitivity analysis evaluating the interaction between baseline Lp(a) and LDL-C levels with diabetes for ASCVD risk and performed similar analyses to those previously mentioned stratified by diabetes status. Participants from JUPITER were excluded from this analysis because there were no individuals with diabetes, and history of vascular disease was not adjusted for in the stratified analyses because there were no participants without vascular disease and without diabetes. An analysis also stratifying by history of vascular disease was performed to account for this. We also performed a sensitivity analysis evaluating each study individually.

To evaluate the continuous association between baseline Lp(a) and ASCVD risk, we modeled the relative risk increase (expressed as a hazard ratio) for ASCVD depending on an individual’s baseline Lp(a) level, compared with a reference level of 5 mg/dL, separately for patients on placebo or statin using generalized additive Cox models.^25^ A key advantage of generalized additive models is their flexibility in effect modeling of continuous covariates without imposing a specific parametric form, whereas smoothing is enforced (and overfitting avoided) because of penalization. The models were adjusted for the covariates listed previously, included the interaction between baseline Lp(a) and LDL-C, and were stratified by trial and treatment assignment.

To investigate whether baseline Lp(a) affects the association between baseline LDL-C and ASCVD risk, we used Cox proportional hazards models with an interaction term for baseline Lp(a) and LDL-C, adjusted for the covariates listed previously, stratified by trial and treatment assignment, and included a study-specific random-effect term for Lp(a). This analysis was performed in the overall cohort and separately for those assigned placebo or statin. As a sensitivity analysis, the association between baseline LDL-C level and ASCVD risk was evaluated using LDL-C corrected for the component of Lp(a) cholesterol. A range of correction factors from 20% to 45% was used by subtracting 20% to 45% of Lp(a) mass from LDL-C.

We evaluated the association between categories of baseline Lp(a) and statin-achieved LDL-C with ASCVD risk in statin-treated individuals. In this analysis, participants were categorized by baseline Lp(a) dichotomized at 50 mg/dL (≈125 nmol/L) and by achieved LDL-C quartiles. A threshold of 50 mg/dL for Lp(a) was chosen, as current guidelines define levels above this threshold as elevated or high risk.^1,6^ Again, models were adjusted for the covariates listed previously and stratified by trial. We also categorized individuals by baseline Lp(a) level and (study-specific and overall) quartile of absolute change in LDL-C level with statin therapy. We performed multiple sensitivity analyses. We categorized participants based on a study-specific median achieved LDL-C level, by an achieved LDL-C threshold of 100 or 55 mg/dL, or by statin-achieved Lp(a) and LDL-C levels. Observed event rates per 100 person-years were calculated.

All analyses were performed using RStudio (version 2023.06.1; Posit Software). A 2-tailed *P*<0.05 was considered statistically significant.

## RESULTS

### Baseline Characteristics

**Figure 1.**
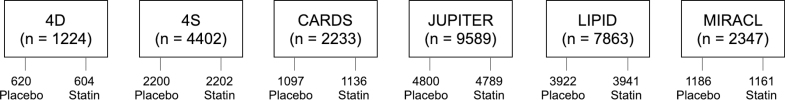
**Study cohort.** The flowchart depicts the number of participants in each trial with available lipoprotein(a) measurements and the number randomized to statin versus placebo. 4D indicates Die Deutsche Diabetes Dialyse; 4S, Scandinavian Simvastatin Survival Study; CARDS, Collaborative Atorvastatin Diabetes Study; JUPITER, Justification for the Use of Statins in Prevention: An Intervention Trial Evaluating Rosuvastatin; LIPID, Long-Term Intervention With Pravastatin in Ischaemic Disease; and MIRACL, Myocardial Ischemia Reduction With Aggressive Cholesterol Lowering.

In total, 29 098 participants from the 6 trials were considered for analysis. After excluding those with missing Lp(a) (n=1190) or LDL-C (n=250) data, there were 27 658 participants remaining. The number of participants per study in the placebo and statin arms is shown in Figure 1. Baseline characteristics for the participants from each trial are shown in Table 1.

**Table 1.**
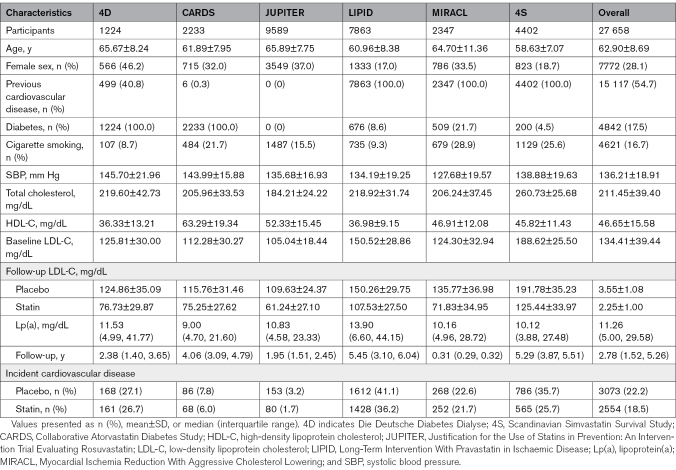
Characteristics of Trial Participants

### Baseline LDL-C and Lp(a) Levels and ASCVD Risk

Baseline Lp(a) and LDL-C levels were both independently associated with ASCVD risk overall (Lp(a) per SD: HR, 1.07 [95% CI, 1.04–1.10], *P*<0.001; LDL-C per SD: HR, 1.06 [95% CI, 1.02–1.10], *P*=0.001). No meaningful difference in these HRs was noted when a range of corrected LDL-C values was used. Among patients assigned to placebo, both were significantly associated with ASCVD risk (Lp(a) per SD: HR, 1.05 [95% CI, 1.01–1.10], *P*=0.013; LDL-C per SD: HR, 1.08 [95% CI, 1.03–1.14], *P*=0.002). Among patients assigned to statin, Lp(a) remained significantly associated with ASCVD risk (HR, per SD: 1.08 [95% CI, 1.04–1.11], *P*<0.001), whereas baseline LDL-C was not significantly associated (HR, per SD: 1.04 [95% CI, 0.98–1.10], *P*=0.170). The multiplicative interaction between Lp(a) and LDL-C in these models in the overall cohort was not significant (*P*=0.170). There was also no significant interaction specifically among women (*P*=0.600) or men (*P*=0.150). There was also no significant interaction between Lp(a) and sex for ASCVD risk (*P*=0.310), with similar risk associated with Lp(a) for women (HR, per SD: 1.07 [95% CI, 1.02–1.11], *P*=0.004) and men (HR, 1.08 [95% CI, 1.04–1.12], *P*<0.001). There was no significant interaction between Lp(a) and LDL-C among those younger than or equal to the median age of 63 years (*P*=0.710). There was a significant interaction among those greater than the median age (*P*=0.009), but this must be interpreted with caution, given age differences between trials.

**Figure 2.**
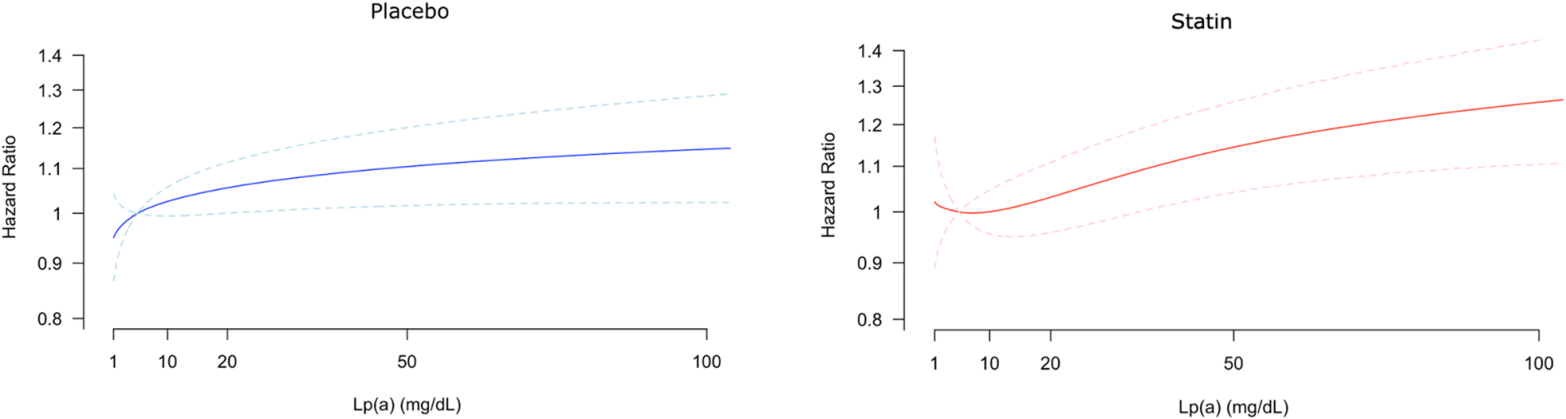
**Association between baseline lipoprotein(a) level and atherosclerotic cardiovascular disease risk in placebo and statin treatment arms.** Atherosclerotic cardiovascular disease risk increases log-linearly with increasing lipoprotein(a) (Lp[a]) levels in both the placebo and statin treatment arms. Hazard ratios were calculated using a reference Lp(a) level of 5 mg/dL. Adjusted for age, sex, history of vascular disease, diabetes, smoking, systolic blood pressure, baseline low-density lipoprotein cholesterol level, and high-density lipoprotein cholesterol level, and stratified by trial and treatment assignment.

ASCVD risk associated with baseline Lp(a) levels in all participants was modeled continuously in multivariable adjusted models, centered at a reference Lp(a) of 5 mg/dL. Above this level, the risk associated with Lp(a) increased in a log-linear fashion in both the placebo and statin treatment groups (Figure 2). The multiplicative interaction between Lp(a) and LDL-C in these models was not significant (*P*=0.125).

Next, the association between baseline LDL-C and ASCVD risk was evaluated by baseline Lp(a) level continuously. The association between LDL-C and ASCVD risk appeared flat, without variation by baseline Lp(a) level in the overall study population. As expected, absolute risk was higher in the placebo group compared with the statin-treated group (Figure S1).

There was no significant interaction between baseline Lp(a) and presence of diabetes for ASCVD risk (*P*=0.910) overall or in the placebo (*P*=0.330) or statin (*P*=0.480) groups. There was a borderline statistically significant interaction for LDL-C and diabetes (*P*=0.056) overall with a stronger association between LDL-C and ASCVD risk among those with diabetes, with similar results among the statin group, but no significant interaction in the placebo group (Table S1). When stratified by previous history of cardiovascular disease, there was no significant interaction between Lp(a) or LDL-C and diabetes among those with history of CVD (Table S2). We also observed a consistent positive association between Lp(a) and LDL-C, evaluated continuously, and ASCVD risk when evaluated by individual study (Table S3).

### Baseline Lp(a), Achieved LDL-C, and ASCVD Risk on Statin Therapy

Statin-treated individuals were categorized by baseline Lp(a) (dichotomized at 50 mg/dL) and achieved LDL-C quartile on statin therapy. Study-specific median baseline LDL-C ranged from 108.2 to 187.5 mg/dL. Median achieved LDL-C ranged from 54.1 to 119.9 mg/dL. Median absolute LDL-C change in response to statin therapy ranged from −39.1 to −67.7 mg/dL, and median percentage LDL-C change ranged from −30.4% to −48.5% (Table 2). Achieved LDL-C quartiles were as follows: quartile 1, <77.34; quartile 2, 77.34 to 109.44; quartile 3, 109.82 to 140.37; and quartile 4, >140.76 mg/dL.

**Table 2.**
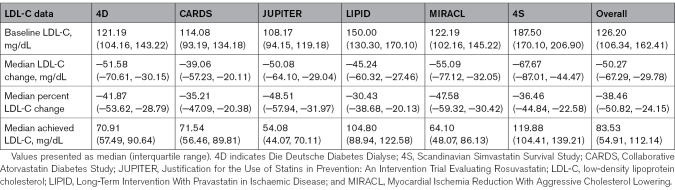
LDL-C Response in Statin Arms of Clinical Trials

**Figure 3.**
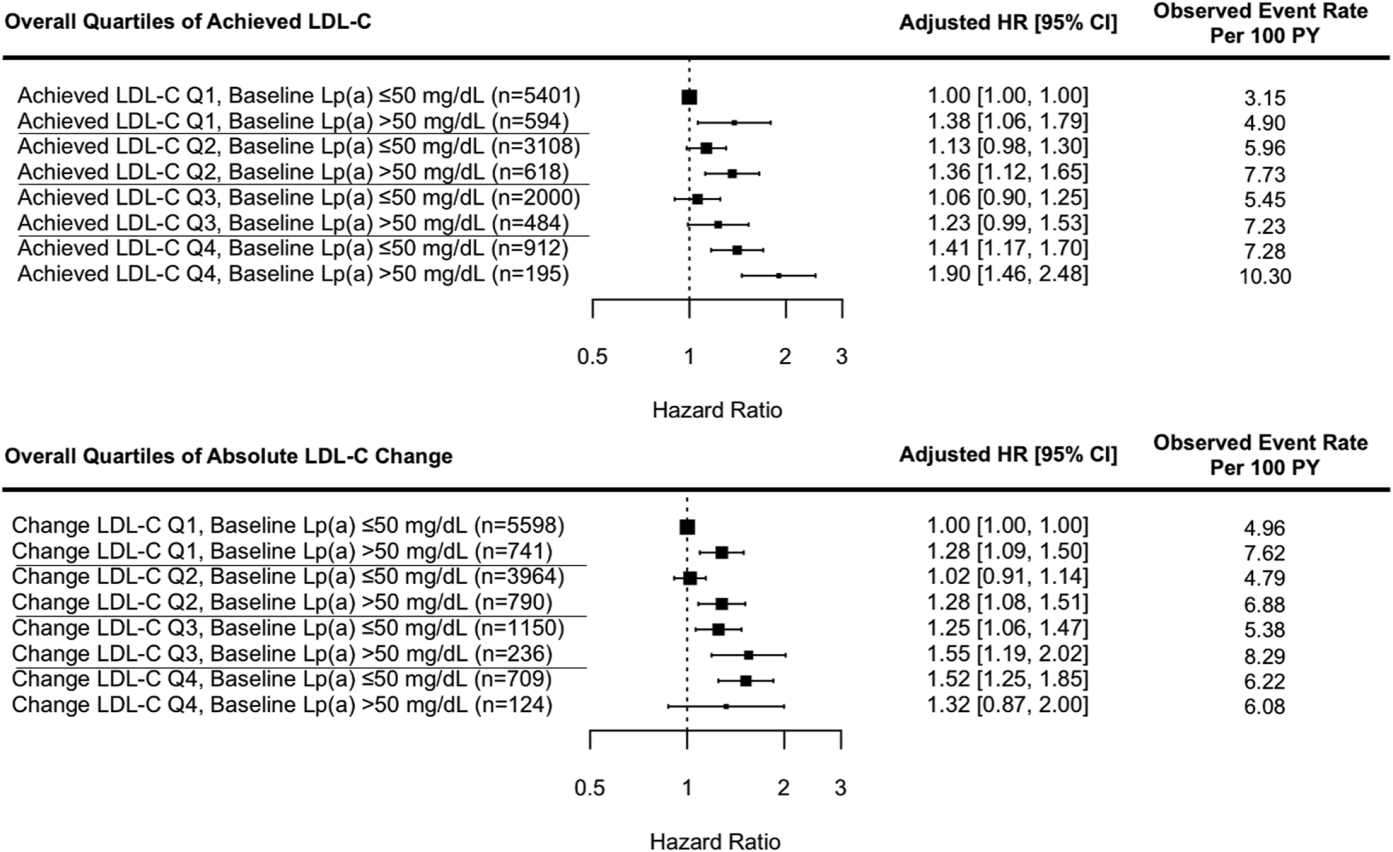
**Joint association between baseline lipoprotein(a) level and achieved low-density lipoprotein cholesterol level or change in low-density lipoprotein cholesterol level and atherosclerotic cardiovascular disease risk in statin users.** Hazard ratio (HR) adjusted for age, sex, history of vascular disease, diabetes, smoking, systolic blood pressure, baseline low-density lipoprotein cholesterol (LDL-C), and high-density lipoprotein cholesterol (HDL-C), and stratified by trial. Achieved LDL-C quartiles in mg/dL: quartile 1, 3.09–76.95; quartile 2, 77.34 to 109.44; quartile 3, 109.82 to 140.37; quartile 4, >140.76). Absolute change from baseline in LDL-C quartiles in mg/dL: quartile 1, −192.6 to −52.09; quartile 2, −52.09 to −18.56; quartile 3, −18.56 to 5.99; quartile 4, 5.99 to 115.24. PY indicates person-years.

Compared with Lp(a) ≤50 mg/dL and achieved LDL-C in the first quartile, increased risk was observed when achieved LDL-C was in the fourth quartile with Lp(a) ≤50 mg/dL (HR, 1.41 [95% CI, 1.17–1.70]), when Lp(a) was >50 mg/dL with LDL-C in the first quartile (HR, 1.38 [95% CI, 1.06–1.79]), and particularly when Lp(a) was >50 mg/dL and LDL-C was in the fourth quartile (HR, 1.90 [95% CI, 1.46–2.48]). A similar trend of increasing observed event rates with higher LDL-C and Lp(a) levels was observed (Figure 3). Similar results were observed when follow-up Lp(a) was used instead of baseline Lp(a) with higher HRs overall when Lp(a) was elevated (for Lp(a) >50 mg/dL and achieved LDL-C in the fourth quartile, HR was 2.13 [95% CI, 1.65–2.75]). Similar results were also observed when categorizing achieved LDL-C using study-specific quartiles (data not shown), when using the study-specific median, or with an LDL-C threshold of 100 mg/dL (Figure S2). Results were also consistent when comparing those with Lp(a) >50 mg/dL and achieved LDL-C ≤55 mg/dL compared with those with Lp(a) ≤50 mg/dL and achieved LDL-C ≤55 mg/dL (HR, 1.39 [95% CI, 0.86–2.24]), although power was limited by the small number of participants (n=184).

Statin-treated individuals were also categorized by baseline Lp(a) level and quartile of absolute change in LDL-C. Among those with Lp(a) ≤50 mg/dL, there was an increase in ASCVD risk in those with less absolute change in LDL-C (HR, 1.52 [95% CI, 1.25–1.85] for fourth quartile of LDL-C change). However, in those with Lp(a) >50 mg/dL, ASCVD risk was increased across categories, even with the greatest LDL-C change (HR, 1.28 [95% CI, 1.09–1.50] for first quartile of LDL-C change; Figure 3). Similar results were seen when study-specific quartiles of relative change in LDL-C were used (Figure S3).

## DISCUSSION

In this large participant-level meta-analysis of statin trials, it was observed that baseline Lp(a) level was associated with cardiovascular risk continuously and independently of baseline LDL-C, elevated Lp(a) level was associated with increased risk even when LDL-C was treated to low levels with statin therapy (both by achieved levels and absolute change), and the greatest risk occurred when both Lp(a) and LDL-C levels were elevated. Taken together, these findings demonstrate that Lp(a) and LDL-C are independent and additive for cardiovascular risk, even on statin treatment, and LDL-C reduction does not completely offset Lp(a)-mediated risk.

Our study has important clinical implications for the management of patients with elevated Lp(a) level. First, there does not appear to be sufficient evidence that reducing LDL-C can fully offset the risk associated with Lp(a), both when assessed by achieved levels and absolute change, emphasizing that both are important targets for risk reduction requiring tailored management approaches, especially with the advent of targeted therapies.^26^ Paradigms for lowering LDL-C level to offset Lp(a)-mediated risk have been proposed previously, including in the Lp(a) consensus statement from the European Atherosclerosis Society, which provides estimates for the degree of LDL-C lowering needed to offset risk at a given Lp(a) level based on age of initiation, up to age 80 years.^3^ Our study has a shorter time of LDL-C lowering, with a median follow-up of 2.8 years. However, even in those achieving the lowest LDL-C concentrations (quartile 1: <77.34 mg/dL), there was still a 38% increased relative risk in those with Lp(a) >50 mg/dL compared with those with ≤50 mg/dL, suggesting that Lp(a)-mediated risk cannot be fully offset by lowering LDL-C level, and that these are independent pathways for atherothrombosis. In addition, with the greatest degree of absolute change in LDL-C level (decrease of 52 to 193 mg/dL), there was still greater overall risk in those with Lp(a) >50 mg/dL compared with those with Lp(a) ≤50 mg/dL. Consistent with these data, it was demonstrated in the UK Biobank that apolipoprotein B100 also does not explain the ASCVD risk mediated by Lp(a), but that it does explain the risk mediated by LDL-C.^27^ This further implies that other components of Lp(a), such as its content of oxidized phospholipids,^28^ antifibrinolytic effects,^29^ or potential pro-platelet effects,^30^ may be more important in mediating clinical manifestations.

Second, our findings suggest that both LDL-C and Lp(a) levels are important and independent targets for reducing cardiovascular risk. In analyses of PCSK9i trials, the greatest benefit was observed when Lp(a) levels were elevated. Given the modest Lp(a)-lowering achieved with PCSK9 inhibitors, and our study demonstrating that LDL-C lowering alone cannot eliminate Lp(a)-mediated risk, these findings suggest that both LDL-C and Lp(a) are important for cardiovascular risk. In an analysis of FOURIER (Further Cardiovascular Outcomes Research With PCSK9 Inhibition in Subjects With Elevated Risk), which compared the PCSK9i evolocumab with placebo on a background of moderate to high-intensity statin, the lowest event rate was observed when achieved Lp(a) was ≤50 mg/dL and LDL-C was ≤70 mg/dL (5.88%). Increased risk was observed when LDL-C was elevated >70 mg/dL (7.37%) or when Lp(a) was elevated >50 mg/dL (7.46%), with the greatest risk observed when both were elevated (8.86%).^9^ In ODYSSEY OUTCOMES (Evaluation of Cardiovascular Outcomes After an Acute Coronary Syndrome During Treatment With Alirocumab), which compared the PCSK9i alirocumab with placebo on a background of high-intensity or maximum-tolerated statin, baseline Lp(a) quartile strongly predicted risk of cardiovascular events in the placebo group and predicted cardiovascular event reduction with alirocumab.^10^ The latter observation was extended in a post hoc subgroup analysis of trial participants with baseline LDL-C levels near 70 mg/dL, in whom a concurrent baseline Lp(a) level above the median (13.7 mg/dL) was associated with treatment benefit of alirocumab.^31^ This suggests that in patients with nominally controlled LDL-C, the greatest benefit to PCSK9i occurs with higher Lp(a) levels, likely due to a combination of additional LDL-C lowering or modest Lp(a) lowering.

Third, the demonstration of a continuous association between Lp(a) level and cardiovascular risk calls into question the current paradigm of using a threshold of 50 mg/dL as recommended in some guidelines,^1^ which has important implications for risk assessment. The 50-mg/dL threshold was based on the 80th percentile of a Danish population of 6000 individuals, and was not necessarily defined by pathophysiologic risk of ASCVD events.^32^ It is also well established that the prevalence of elevated Lp(a) level differs markedly by race and ethnicity, so that using a single cutoff may not be broadly representative globally.^24^ It also does not discriminate risk on the basis of whether individuals are categorized as being treated for primary or secondary prevention. Unlike for LDL-C, in which the relative risk reduction appears to be similar in primary and secondary prevention,^33^ this will not be established for Lp(a) until the completion of cardiovascular outcomes trials evaluating targeted Lp(a) therapeutics. We demonstrate in a large meta-analysis of randomized trials that Lp(a) and LDL-C are independent and additive for cardiovascular risk as the risks associated with each were not modified by the other, and multiplicative interaction tests were nonsignificant. These findings emphasize the need for more widespread Lp(a) testing and for aggressive management of patients with elevated Lp(a) level. Further study is needed to understand the economic implications of this, particularly given the high prevalence of elevated Lp(a) levels and potential for novel therapies soon. However, Lp(a) testing is relatively inexpensive, and only needs to be performed once in most individuals.^34^ In addition, given the high burden of disease associated with elevated Lp(a) level, particularly premature cardiovascular disease, it is possible that the upfront investment in more aggressive management will reduce downstream costs associated with worse cardiovascular outcomes.

Previous studies have sought to address the relationship between Lp(a) and LDL-C levels for cardiovascular risk. In a similar meta-analysis of 7 statin trials, the association between Lp(a) level and cardiovascular risk appeared to increase linearly and independently of LDL-C level when evaluated at specific Lp(a) cut points. This study also demonstrated that the independent risk associated with Lp(a) level persisted in statin-treated individuals.^11^ Our study adds to this previous work by demonstrating that Lp(a) and LDL-C levels are independent and additive predictors of cardiovascular risk. Whereas the previous study evaluated Lp(a)-mediated risk specifically, we evaluated the implications of LDL-C lowering on global cardiovascular risk in the context of elevated Lp(a) level, demonstrating that even with the lowest achieved LDL-C levels (both absolutely and relatively), overall risk remains increased in those with elevated Lp(a) level, and potent LDL-C lowering does not appear to completely offset the risk associated with elevated Lp(a) level.

In an observational study from MESA (Multi-Ethnic Study of Atherosclerosis), participants were categorized similarly by Lp(a) level above or below 50 mg/dL and LDL-C level above or below 100 mg/dL. Over a mean follow-up of 13.4 years, there was increased risk when Lp(a) level alone was elevated (HR, 1.83 [95% CI, 1.02–3.27]) compared with when Lp(a) and LDL-C levels were both lower. MESA used baseline Lp(a) and LDL-C results, excluded individuals on lipid-lowering therapy, and had only a small number of individuals with isolated elevated Lp(a) level (n=130 among the total population of 4585).^7^ In addition, the risk of Lp(a) level and very elevated LDL-C level appears to be additive, with higher risk when elevated Lp(a) level and familial hypercholesterolemia are both present.^35^

A recent study of individuals from pooled cohort studies observed a significant interaction between Lp(a) level and diabetes, with increased ASCVD risk associated with Lp(a) level among those with diabetes.^36^ However, we did not find an association between Lp(a) level and diabetes for ASCVD risk. This may be due to significant differences in the study designs, as the previous study was conducted in individuals free of baseline ASCVD and pooled data from multiple observational prospective cohort studies. In contrast, in the current study, 55% of individuals had previous cardiovascular disease, and this was conducted as a meta-analysis of randomized controlled trials. There was no significant interaction between Lp(a) level and diabetes among those with previous cardiovascular disease, but we were unable to test this among those without a history of cardiovascular disease, as there were no participants in this group without diabetes. Another previous study observed that Lp(a) level was only associated with increased risk in women when total cholesterol level was >220 mg/dL among participants in the Women’s Health Study.^37^ However, in our study, we observed that there was no significant interaction between Lp(a) and LDL-C levels among women or men, and there was no significant interaction between Lp(a) level and sex for ASCVD risk, with similar risk associated with Lp(a) level among both men and women. We did not have sufficient sample size to perform analyses stratified by sex because most trial participants were men. Together, these results do not suggest that the association between Lp(a) level and ASCVD risk is particularly modified by LDL-C level among women. This aligns with a recent analysis from ODYSSEY OUTCOMES: in participants with mean LDL-C level <90 mg/dL, Lp(a) level was similarly associated with cardiovascular risk in men and women.^38^

An important limitation in interpreting the relationship between LDL-C and Lp(a) levels for cardiovascular risk is that the reporting of LDL-C level, whether estimated or measured directly, contains Lp(a) cholesterol content, which can contribute ≈15 to 30 mg/dL to measured “LDL-C” in patients with elevated Lp(a) level.^39^ In fact, in an analysis from the Lipoprotein Studies Collaboration in a similar data set as this study, it was demonstrated that baseline LDL-C level was no longer predictive of major adverse cardiovascular events if its Lp(a) cholesterol content was corrected mathematically using a range of 20% to 45% content of Lp(a) that is cholesterol by weight.^40^ It has subsequently been recommended that correction factors for Lp(a) cholesterol not be used, as Lp(a) cholesterol relative to Lp(a) mass may range from 6% to 57%.^41^ Therefore, direct quantitation is required to determine Lp(a) cholesterol content properly, and subsequently correct the LDL-C level. In a sensitivity analysis, we did not find a significantly different association between LDL-C level and ASCVD risk when examining a range of factors to correct for Lp(a) cholesterol.

The findings of this study should be interpreted in the context of its limitations. The studies included are heterogeneous, with different populations, baseline LDL-C levels, and strength of statin therapy used. There was also significant variability in the length of follow-up of the studies. The 4D study is an outlier among placebo-controlled studies of statins on cardiovascular outcomes, because no benefit of statin treatment was observed. In addition, the methods for measurement of Lp(a) varied between studies, and most studies used mass assays (in mg/dL) rather than molar concentration assays (nmol/L); those measured in nmol/L were converted to mg/dL, which may lead to an imprecise measurement of actual Lp(a) levels. However, the associations between Lp(a) and LDL-C levels with ASCVD risk appeared similar across studies when evaluated individually. In addition, a recent study suggested that, at a trial level, units of measurement do not make a meaningful difference for event prediction.^42^ It is also possible that different results would be obtained with more potent LDL-C lowering, such as with combination therapy or with PCSK9i. However, we did not observe that LDL-C lowering offset risk in those with elevated Lp(a) level even in the lowest quartile of achieved LDL-C levels. The included trials were not designed to evaluate Lp(a) level specifically and were not enriched with individuals with higher Lp(a) levels. The LDL-C measure used in the current analysis was the laboratory-reported LDL-C level, which also contains the Lp(a)-C content. Correcting LDL-C for a range of factors to estimate Lp(a) cholesterol level did not meaningfully change the association between LDL-C level and risk, but ultimately, methods to measure and report the value of LDL-C level without its Lp(a) content may be needed to further evaluate their respective risk and to optimally manage individuals with elevated LDL-C and Lp(a) levels, particularly with the anticipated availability of targeted medical therapy for elevated Lp(a) level. The lack of a significant interaction between Lp(a) and LDL-C levels may also be due to limited power or model misspecification. There was also insufficient racial and ethnic representation to conduct analyses stratified by race or ethnicity or using race-specific percentiles for Lp(a) level, as the majority of participants were White. Additional potential confounders, such as diet, physical activity, and genetic factors, were not available and thus could not be adjusted for. However, Lp(a) level is primarily genetically determined and is only modestly affected by lifestyle factors,^43^ and accounting for genetic factors does not appear to improve risk prediction in addition to Lp(a) levels.^44^

In this meta-analysis of statin trials, Lp(a) and LDL-C levels were independent and additive cardiovascular risk factors, and potent LDL-C level reduction did not offset Lp(a)-mediated risk. Our findings emphasize the need for testing and incorporation of Lp(a) into cardiovascular risk assessment. Future trials will elucidate the role of targeted Lp(a) therapeutics for cardiovascular risk reduction in the context of LDL-C levels.

## ARTICLE INFORMATION

### Acknowledgments

The authors thank the participants, investigators, and staff of each of the included trials for their contributions.

### Sources of Funding

This work was funded by Novartis through a grant to Drs Tsimikas and Bhatia to conduct the meta-analysis. Additional support was provided through grants 1KL2TR001444 and 1K08HL166962 (Dr Bhatia) and National Institutes of Health R01 HL159156 and HL170224 (Dr Tsimikas).

### Disclosures

Dr Bhatia received consulting fees from Abbott, Arrowhead, Kaneka Medical, and Novartis Pharmaceuticals. Dr Tsimikas is a coinventor and receives royalties from patents owned by University of California, San Diego and is a cofounder and has an equity interest in Kleanthi Diagnostics, LLC, and has a dual appointment at University of California, San Diego and Ionis Pharmaceuticals. The terms of this arrangement have been reviewed and approved by the University of California, San Diego in accordance with its conflict of interest policies. Drs Lesogor and Wandel, and K. Bailey are employees of Novartis. Dr Wandel holds stocks of Novartis, Alcon, and Sandoz. A. Tonkin has received consulting fees from Novartis and is on the DMC of the ORION-4 study. Dr Ridker has received institutional research grant support from Kowa, Novartis, Amarin, Pfizer, Esperion, Novo Nordisk, and the National Heart, Lung, and Blood Institute; has served as a consultant to Novartis, Flame, Agepha, Ardelyx, AstraZeneca, Janssen, Civi Biopharm, GSK, SOCAR, Novo Nordisk, Health Outlook, Montai Health, Eli Lilly, New Amsterdam, Boehringer-Ingelheim, RTI, Zomagen, Cytokinetics, Horizon Therapeutics, and Cardio Therapeutics; has minority shareholder equity positions in Uppton, Bitteroot Bio, and Angiowave; and receives compensation for service on the Peter Munk advisory board (University of Toronto), the Leducq Foundation, Paris FR, and the Baim Institute. Dr Willeit reports consulting fees from Novartis Pharmaceuticals. Dr Colhoun reports grants from IQVIA, JDRF, Chief Scientist Office, Diabetes UK, MRC (UKRI), and EU Commission; speaker bureau fees from Novo Nordisk; advisory roles with Novo Nordisk and Bayer AG; and is a shareholder in Roche Pharmaceuticals and Bayer AG. The other coauthors have nothing to disclose.

### Supplemental Material

Figures S1–S3

Tables S1–S3

## References

[1] Grundy SM Stone NJ Bailey AL Beam C Birtcher KK Blumenthal RS Braun LT de Ferranti S Faiella-Tommasino J Forman DE. 2018 AHA/ACC/AACVPR/AAPA/ABC/ACPM/ADA/AGS/APhA/ASPC/NLA/PCNA guideline on the management of blood cholesterol: a report of the American College of Cardiology/American Heart Association task force on clinical practice guidelines. J Am Coll Cardiol. 2019;73:e285–e350. doi: 10.1016/j.jacc.2018.11.003 30423393 10.1016/j.jacc.2018.11.003

[2] Bhatia HS Wilkinson MJ. Lipoprotein(a): evidence for role as a causal risk factor in cardiovascular disease and emerging therapies. J Clin Med. 2022;11:6040. doi: 10.3390/jcm11206040 36294361 10.3390/jcm11206040 PMC9604626

[3] Kronenberg F Mora S Stroes ESG Ference BA Arsenault BJ Berglund L Dweck MR Koschinsky M Lambert G Mach F. Lipoprotein(a) in atherosclerotic cardiovascular disease and aortic stenosis: a European Atherosclerosis Society consensus statement. Eur Heart J. 2022;43:3925–3946. doi: 10.1093/eurheartj/ehac361 36036785 10.1093/eurheartj/ehac361 PMC9639807

[4] Pearson GJ Thanassoulis G Anderson TJ Barry AR Couture P Dayan N Francis GA Genest J Grégoire J Grover SA. 2021 Canadian Cardiovascular Society guidelines for the management of dyslipidemia for the prevention of cardiovascular disease in adults. Can J Cardiol. 2021;37:1129–1150. doi: 10.1016/j.cjca.2021.03.016 33781847 10.1016/j.cjca.2021.03.016

[5] Mach F Baigent C Catapano AL Koskinas KC Casula M Badimon L Chapman MJ De Backer GG Delgado V Ference BA. 2019 ESC/EAS guidelines for the management of dyslipidaemias: lipid modification to reduce cardiovascular risk. Eur Heart J. 2020;41:111–188. doi: 10.1093/eurheartj/ehz455 31504418 10.1093/eurheartj/ehz455

[6] Koschinsky ML Bajaj A Boffa MB Dixon DL Ferdinand KC Gidding SS Gill EA Jacobson TA Michos ED Safarova MS. A focused update to the 2019 NLA scientific statement on use of lipoprotein(a) in clinical practice. J Clin Lipidol. 2024;18:e308–e319. doi: 10.1016/j.jacl.2024.03.001 38565461 10.1016/j.jacl.2024.03.001

[7] Rikhi R Hammoud A Ashburn N Snavely AC Michos ED Chevli P Tsai MY Herrington D Shapiro MD. Relationship of low-density lipoprotein-cholesterol and lipoprotein(a) to cardiovascular risk: the Multi-Ethnic Study of Atherosclerosis (MESA). Atherosclerosis. 2022;363:102–108. doi: 10.1016/j.atherosclerosis.2022.10.004 36253168 10.1016/j.atherosclerosis.2022.10.004 PMC9964094

[8] Madsen CM Kamstrup PR Langsted A Varbo A Nordestgaard BG. Lipoprotein(a)-lowering by 50 mg/dL (105 nmol/L) may be needed to reduce cardiovascular disease 20% in secondary prevention: a population-based study. Arterioscler Thromb Vasc Biol. 2020;40:255–266. doi: 10.1161/ATVBAHA.119.312951 31578080 10.1161/ATVBAHA.119.312951

[9] O’Donoghue ML Fazio S Giugliano RP Stroes ESG Kanevsky E Gouni-Berthold I Im K Lira Pineda A Wasserman SM Češka R. Lipoprotein(a), PCSK9 inhibition, and cardiovascular risk. Circulation. 2019;139:1483–1492. doi: 10.1161/CIRCULATIONAHA.118.037184 30586750 10.1161/CIRCULATIONAHA.118.037184

[10] Szarek M Bittner VA Aylward P Baccara-Dinet M Bhatt DL Diaz R Fras Z Goodman SG Halvorsen S Harrington RA; ODYSSEY OUTCOMES Investigators. Lipoprotein(a) lowering by alirocumab reduces the total burden of cardiovascular events independent of low-density lipoprotein cholesterol lowering: ODYSSEY OUTCOMES trial. Eur Heart J. 2020;41:4245–4255. doi: 10.1093/eurheartj/ehaa649 33051646 10.1093/eurheartj/ehaa649 PMC7724642

[11] Willeit P Ridker PM Nestel PJ Simes J Tonkin AM Pedersen TR Schwartz GG Olsson AG Colhoun HM Kronenberg F. Baseline and on-statin treatment lipoprotein(a) levels for prediction of cardiovascular events: individual patient-data meta-analysis of statin outcome trials. Lancet. 2018;392:1311–1320. doi: 10.1016/S0140-6736(18)31652-0 30293769 10.1016/S0140-6736(18)31652-0

[12] Khera AV Everett BM Caulfield MP Hantash FM Wohlgemuth J Ridker PM Mora S. Lipoprotein(a) concentrations, rosuvastatin therapy, and residual vascular risk: an analysis from the JUPITER Trial (Justification for the Use of Statins in Prevention: an Intervention Trial Evaluating Rosuvastatin). Circulation. 2014;129:635–642. doi: 10.1161/CIRCULATIONAHA.113.004406 24243886 10.1161/CIRCULATIONAHA.113.004406 PMC3946056

[13] Albers JJ Slee A O’Brien KD Robinson JG Kashyap ML Kwiterovich PO Jr Xu P Marcovina SM. Relationship of apolipoproteins A-1 and B, and lipoprotein(a) to cardiovascular outcomes: the AIM-HIGH trial (Atherothrombosis Intervention in Metabolic Syndrome with Low HDL/High Triglyceride and Impact on Global Health Outcomes). J Am Coll Cardiol. 2013;62:1575–1579. doi: 10.1016/j.jacc.2013.06.051 23973688 10.1016/j.jacc.2013.06.051 PMC3800510

[14] Nestel PJ Barnes EH Tonkin AM Simes J Fournier M White HD Colquhoun DM Blankenberg S Sullivan DR. Plasma lipoprotein(a) concentration predicts future coronary and cardiovascular events in patients with stable coronary heart disease. Arterioscler Thromb Vasc Biol. 2013;33:2902–2908. doi: 10.1161/ATVBAHA.113.302479 24092750 10.1161/ATVBAHA.113.302479

[15] Tsimikas S Gordts P Nora C Yeang C Witztum JL. Statin therapy increases lipoprotein(a) levels. Eur Heart J. 2020;41:2275–2284. doi: 10.1093/eurheartj/ehz310 31111151 10.1093/eurheartj/ehz310

[16] Efficacy and safety of LDL-lowering therapy among men and women: meta-analysis of individual data from 174 000 participants in 27 randomised trials. Lancet. 2015;385:1397–1405. doi: https://doi.org/10.1016/S0140-6736(14)61368-4 25579834 10.1016/S0140-6736(14)61368-4

[17] Tsimikas S Moriarty PM Stroes ES. Emerging RNA therapeutics to lower blood levels of Lp(a): JACC focus seminar 2/4. J Am Coll Cardiol. 2021;77:1576–1589. doi: 10.1016/j.jacc.2021.01.051 33766265 10.1016/j.jacc.2021.01.051

[18] Krane V Schmidt KR Gutjahr-Lengsfeld LJ Mann JF März W Swoboda F Wanner C; 4D Study Investigators (the German Diabetes and Dialysis Study Investigators). Long-term effects following 4 years of randomized treatment with atorvastatin in patients with type 2 diabetes mellitus on hemodialysis. Kidney Int. 2016;89:1380–1387. doi: 10.1016/j.kint.2015.12.033 26924051 10.1016/j.kint.2015.12.033

[19] Randomised trial of cholesterol lowering in 4444 patients with coronary heart disease: the Scandinavian Simvastatin Survival Study (4S). Lancet. 1994;344:1383–1389. 7968073

[20] Colhoun HM Betteridge DJ Durrington PN Hitman GA Neil HA Livingstone SJ Thomason MJ Mackness MI Charlton-Menys V Fuller JH; CARDS Investigators. Primary prevention of cardiovascular disease with atorvastatin in type 2 diabetes in the Collaborative Atorvastatin Diabetes Study (CARDS): multicentre randomised placebo-controlled trial. Lancet. 2004;364:685–696. doi: 10.1016/S0140-6736(04)16895-5 15325833 10.1016/S0140-6736(04)16895-5

[21] Ridker PM Danielson E Fonseca FA Genest J Gotto AM Jr Kastelein JJ Koenig W Libby P Lorenzatti AJ MacFadyen JG; JUPITER Study Group. Rosuvastatin to prevent vascular events in men and women with elevated C-reactive protein. N Engl J Med. 2008;359:2195–2207. doi: 10.1056/NEJMoa0807646 18997196 10.1056/NEJMoa0807646

[22] Long-Term Intervention with Pravastatin in Ischaemic Disease (LIPID) Study Group. Prevention of cardiovascular events and death with pravastatin in patients with coronary heart disease and a broad range of initial cholesterol levels. N Engl J Med. 1998;339:1349–1357. doi: 10.1056/nejm199811053391902 9841303 10.1056/NEJM199811053391902

[23] Schwartz GG Olsson AG Ezekowitz MD Ganz P Oliver MF Waters D Zeiher A Chaitman BR Leslie S Stern T; Myocardial Ischemia Reduction with Aggressive Cholesterol Lowering (MIRACL) Study Investigators. Effects of atorvastatin on early recurrent ischemic events in acute coronary syndromes: the MIRACL study: a randomized controlled trial. JAMA. 2001;285:1711–1718. doi: 10.1001/jama.285.13.1711 11277825 10.1001/jama.285.13.1711

[24] Patel AP Wang M Pirruccello JP Ellinor PT Ng K Kathiresan S Khera AV. Lp(a) (lipoprotein[a]) concentrations and incident atherosclerotic cardiovascular disease: new insights from a large national biobank. Arterioscler Thromb Vasc Biol. 2021;41:465–474. doi: 10.1161/atvbaha.120.315291 33115266 10.1161/ATVBAHA.120.315291 PMC7769893

[25] Hastie TJ Tibshirani RJ. Generalized Additive Models. Chapman and Hall; 1990. 10.1177/096228029500400302 8548102

[26] Tsimikas S Karwatowska-Prokopczuk E Gouni-Berthold I Tardif JC Baum SJ Steinhagen-Thiessen E Shapiro MD Stroes ES Moriarty PM Nordestgaard BG; AKCEA-APO(a)-LRx Study Investigators. Lipoprotein(a) reduction in persons with cardiovascular disease. N Engl J Med. 2020;382:244–255. doi: 10.1056/NEJMoa1905239 31893580 10.1056/NEJMoa1905239

[27] Trinder M Zekavat SM Uddin MM Pampana A Natarajan P. Apolipoprotein B is an insufficient explanation for the risk of coronary disease associated with lipoprotein(a). Cardiovasc Res. 2021;117:1245–1247. doi: 10.1093/cvr/cvab060 33629108 10.1093/cvr/cvab060 PMC8064427

[28] Tsimikas S Witztum JL. Oxidized phospholipids in cardiovascular disease. Nat Rev Cardiol. 2023;21:170–191. doi: 10.1038/s41569-023-00937-4 37848630 10.1038/s41569-023-00937-4

[29] Boffa MB. Beyond fibrinolysis: The confounding role of Lp(a) in thrombosis. Atherosclerosis. 2022;349:72–81. doi: 10.1016/j.atherosclerosis.2022.04.009 35606079 10.1016/j.atherosclerosis.2022.04.009

[30] Bhatia HS Becker RC Leibundgut G Patel M Lacaze P Tonkin A Narula J Tsimikas S. Lipoprotein(a), platelet function and cardiovascular disease. Nat Rev Cardiol. 2023;21:299–311. doi: 10.1038/s41569-023-00947-2 37938756 10.1038/s41569-023-00947-2 PMC11216952

[31] Schwartz GG Szarek M Bittner VA Diaz R Goodman SG Jukema JW Landmesser U López-Jaramillo P Manvelian G Pordy R; ODYSSEY Outcomes Committees and Investigators. Lipoprotein(a) and benefit of PCSK9 inhibition in patients with nominally controlled LDL cholesterol. J Am Coll Cardiol. 2021;78:421–433. doi: 10.1016/j.jacc.2021.04.102 34325831 10.1016/j.jacc.2021.04.102 PMC8822604

[32] Nordestgaard BG Chapman MJ Ray K Borén J Andreotti F Watts GF Ginsberg H Amarenco P Catapano A Descamps OS; European Atherosclerosis Society Consensus Panel. Lipoprotein(a) as a cardiovascular risk factor: current status. Eur Heart J. 2010;31:2844–2853. doi: 10.1093/eurheartj/ehq386 20965889 10.1093/eurheartj/ehq386 PMC3295201

[33] Baigent C Blackwell L Emberson J Holland LE Reith C Bhala N Peto R Barnes EH Keech A Simes J; Cholesterol Treatment Trialists’ Collaboration. Efficacy and safety of more intensive lowering of LDL cholesterol: a meta-analysis of data from 170,000 participants in 26 randomised trials. Lancet. 2010;376:1670–1681. doi: 10.1016/S0140-6736(10)61350-5 21067804 10.1016/S0140-6736(10)61350-5 PMC2988224

[34] Rendler J Murphy M Yeang C. Lipoprotein(a) is a prevalent yet vastly underrecognized risk factor for cardiovascular disease. Health Care Curr Rev. 2024;12:397. 38525410 PMC10959503

[35] Hedegaard BS Bork CS Kaltoft M Klausen IC Schmidt EB Kamstrup PR Langsted A Nordestgaard BG. Equivalent impact of elevated lipoprotein(a) and familial hypercholesterolemia in patients with atherosclerotic cardiovascular disease. J Am Coll Cardiol. 2022;80:1998–2010. doi: 10.1016/j.jacc.2022.09.021 36396201 10.1016/j.jacc.2022.09.021

[36] Wong ND Fan W Hu X Ballantyne C Hoodgeveen RC Tsai MY Browne A Budoff MJ. Lipoprotein(a) and long-term cardiovascular risk in a multi-ethnic pooled prospective cohort. J Am Coll Cardiol. 2024;83:1511–1525. doi: 10.1016/j.jacc.2024.02.031 38631771 10.1016/j.jacc.2024.02.031

[37] Cook NR Mora S Ridker PM. Lipoprotein(a) and cardiovascular risk prediction among women. J Am Coll Cardiol. 2018;72:287–296. doi: 10.1016/j.jacc.2018.04.060 30012322 10.1016/j.jacc.2018.04.060 PMC6277983

[38] Bittner VA Schwartz GG Bhatt DL Chua T De Silva HA Diaz R Goodman SG Harrington RA Jukema JW McGinniss J. Alirocumab and cardiovascular outcomes according to sex and lipoprotein(a) after acute coronary syndrome: ODYSSEY OUTCOMES. J Clin Lipidol. 18:e548–e561. doi: 10.1016/j.jacl.2024.04.122 10.1016/j.jacl.2024.04.122 38960812

[39] Yeang C Karwatowska-Prokopczuk E Su F Dinh B Xia S Witztum Joseph L Tsimikas S. Effect of pelacarsen on lipoprotein(a) cholesterol and corrected low-density lipoprotein cholesterol. J Am Coll Cardiol. 2022;79:1035–1046. doi: 10.1016/j.jacc.2021.12.032 35300814 10.1016/S0735-1097(22)02026-5 PMC8972555

[40] Willeit P Yeang C Moriarty PM Tschiderer L Varvel SA McConnell JP Tsimikas S. Low-density lipoprotein cholesterol corrected for lipoprotein(a) cholesterol, risk thresholds, and cardiovascular events. J Am Heart Assoc. 2020;9:e016318. doi: 10.1161/JAHA.119.016318 33222611 10.1161/JAHA.119.016318 PMC7763787

[41] Yeang C Witztum JL Tsimikas S. Novel method for quantification of lipoprotein(a)-cholesterol: implications for improving accuracy of LDL-C measurements. J Lipid Res. 2021;62:100053. doi: 10.1016/j.jlr.2021.100053 33636163 10.1016/j.jlr.2021.100053 PMC8042377

[42] Szarek M Reijnders E Jukema JW Bhatt DL Bittner VA Diaz R Fazio S Garon G Goodman SG Harrington RA; ODYSSEY OUTCOMES Investigators. Relating lipoprotein(a) concentrations to cardiovascular event risk after acute coronary syndrome: a comparison of 3 tests. Circulation. 2024;149:192–203. doi: 10.1161/CIRCULATIONAHA.123.066398 37632469 10.1161/CIRCULATIONAHA.123.066398 PMC10782942

[43] Enkhmaa B Berglund L. Non-genetic influences on lipoprotein(a) concentrations. Atherosclerosis. 2022;349:53–62. doi: 10.1016/j.atherosclerosis.2022.04.006 35606076 10.1016/j.atherosclerosis.2022.04.006 PMC9549811

[44] Trinder M Uddin MM Finneran P Aragam KG Natarajan P. Clinical utility of lipoprotein(a) and LPA genetic risk score in risk prediction of incident atherosclerotic cardiovascular disease. JAMA Cardiol. 2021;6:287–295. doi: 10.1001/jamacardio.2020.5398 33021622 10.1001/jamacardio.2020.5398 PMC7539232

